# Conservative treatment of asymptomatic ectopic ureterocele: A report of two cases

**DOI:** 10.1002/iju5.12137

**Published:** 2019-12-29

**Authors:** Kazutaka Maruo, Kazuyuki Nishinaka

**Affiliations:** ^1^ Department of Pediatric Urology Hokkaido Medical Center for Child Health and Rehabilitation Sapporo Hokkaido Japan; ^2^ Department of Urology Sapporo Medical University Sapporo Hokkaido Japan

**Keywords:** bladder, hydronephrosis, kidney, ureter, ureterocele

## Abstract

**Introduction:**

There are no established treatments for asymptomatic ectopic ureteroceles, with completely duplicated ureters, during the neonatal period and infancy. However, conservative treatment is sometimes successful.

**Case presentation:**

Two patients were prenatally diagnosed, via ultrasonography, with left hydronephrosis. In each case, magnetic resonance imaging confirmed an ectopic ureterocele accompanying the left, completely duplicated ureter. Prophylactic antibiotics were administered and conservative treatment was started. Currently, one patient is 44 months old and the other is 49 months old; in neither patient has the ureterocele or hydronephrosis been exacerbated.

**Conclusion:**

Patients with (i) a nonfunctional kidney, (ii) mild hydronephrosis or moderate vesicoureteral reflux, (iii) no bladder neck obstruction on urination, and (iv) a Churchill classification ≤Grade II (Churchill classification) may be able to select conservative treatment.

**Abbreviations & Acronyms**
DMSA: dimercaptosuccinic acid
HDN: hydronephrosis
MRI: magnetic resonance imaging
TUI: transurethral incisions
VCUG: voiding cystourethrography
VUR: vesicoureteral reflux

**Keynote message**

We report two cases of asymptomatic ectopic ureteroceles with completely duplicated ureters and their successful conservative treatment. Both patients received continuous antibiotic prophylaxis and showed ureterocele reductions. Patients with Churchill classifications ≤Grade II are candidates for conservative treatment.

## Introduction

The increased use of fetal ultrasound examinations has resulted in an upward trend in the prenatal diagnosis of ectopic ureterocele and in the discovery of fetal HDN.^1^ The objective of ectopic ureterocele treatment is to protect renal function and prevent urinary tract infection; nevertheless, a neonatal or fetal treatment method for asymptomatic ectopic ureteroceles involving complete ureteral duplication has not been established.^2^ In many cases, early TUIs are the first treatment choice because of the risk of the ectopic ureterocele leading to sepsis. However, post‐TUI, there is a 50–100% probability of additional surgery being required due to iatrogenic VUR.^2^, ^3^ We report our experience with conservative treatment and our recommended criteria for patient selection.

## Case presentation

**Figure 1:**
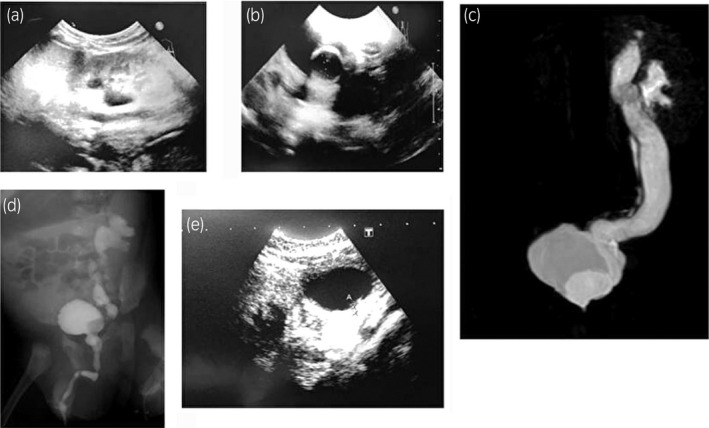
Patient 1. Ultrasonography of the left kidney (a), ultrasonography of the bladder showing a 17‐mm ureterocele (b), MRI showing ectopic ureterocele (c), VCUG showing Grade IV VUR in the left inferior kidney (d), and ultrasonography of the bladder after 38 months of conservative treatment showing reduction of the ureterocele (e).

Patient 1 was a 63 cm, 7.0 kg, 3‐month‐old boy who underwent a postnatal ultrasound examination that revealed mild HDN in the left superior pole, but no HDN in the right kidney. A 17‐mm ureterocele, extending to the bladder neck, was discovered in the bladder (Fig. 1a,b). MRI was performed 2 months after birth and revealed an ectopic ureterocele, with left superior kidney attachment, involving left‐side, complete ureteral duplication (Fig. 1c). At 3 months of age, the patient was referred to our department, and VCUG revealed Grade IV VUR in the left inferior kidney, but no bladder neck obstruction (Fig. 1d); VUR was absent from the left superior or contralateral kidney. Renal scintigraphy, using ^99^mTc‐meso‐2,3‐DMSA, showed a nonfunctional left superior pole.

The patient received a course of prophylactic cefaclor (10–12.5 mg/kg/day), beginning on his first visit. Echocystography, 38 months after his first visit, revealed ureterocele reduction; at 44 months, the patient did not demonstrate ureterocele or HDN worsening (Fig. 1e).

**Figure 2:**
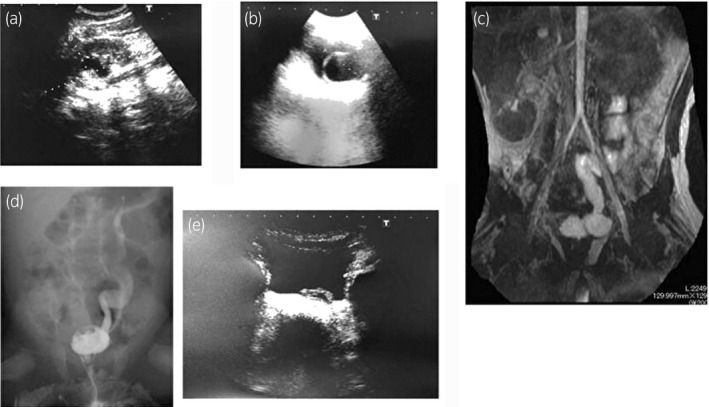
Patient 2. Ultrasonography of the left kidney (a), ultrasonography of the bladder showing an 11‐mm ureterocele (b), MRI showing ectopic ureterocele (c), VCUG showing Grade II VUR in the left superior kidney (d), and ultrasonography of the bladder after 2 months of conservative treatment showing reduction of the ureterocele (e).

Patient 2 was a 6‐day‐old girl (height, 47 cm; weight, 3.0 kg) referred to our department for examination and treatment following a prenatal ultrasound diagnosis of left HDN and ureterocele. Her initial ultrasound revealed mild HDN in the superior pole of the left kidney, but no HDN in the inferior poles of the left or right kidney. An 11‐mm ureterocele, extending to the bladder neck, was discovered in the bladder (Fig. 2a,b). MRI revealed an ectopic ureterocele, with left superior kidney attachment, involving left‐side, complete ureteral duplication (Fig. 2c). VCUG revealed Grade II VUR in the left superior kidney but no bladder neck obstruction (Fig. 2d). Renal scintigraphy, using ^99^mTc‐DMSA, showed a left kidney that was nonfunctional in the superior pole.

The patient received a course of prophylactic amoxicillin (12.5 mg/kg/day), beginning on her first visit. Echocystography, 2 years after the initial visit, showed ureterocele reduction; after 49 months of follow‐up, worsening of the ureterocele or HDN was not observed (Fig. 2e).

## Discussion

We reported these two patients because they illustrate reduction of the ectopic ureterocele during conservative treatment, as well as HDN/hydroureter disappearance. Conservative treatment may be possible in cases where the ureterocele does not obstruct the bladder neck; the Churchill classification,^4^ a method of classifying asymptomatic ectopic ureterocele severity, is useful for selecting such cases.

Treatment of ectopic ureteroceles often requires surgery to avoid the risk of septic shock; hence, conservative treatment is thought to be possible in very few cases. Shankar *et al*. selected follow‐up observation for two patients with prenatal diagnoses of asymptomatic ectopic ureteroceles involving renal pelvis and ureter duplication. Conservative treatment was contingent on three conditions: superior renal function of <10%, an unobstructed inferior kidney or VUR ≤Grade III, and no bladder neck obstruction. During an average follow‐up of 8 years, neither patient developed urinary tract infections or required surgery.^5^ Han *et al*. reported the observation of six patients, without scintigraphic evidence of severe renal obstruction, despite superior renal function. All showed superior urinary tract dilatation improvements; the complicating VUR of the ipsilateral inferior kidney disappeared in five patients.^6^ Direnna *et al*. reported six patients with prenatal diagnoses of asymptomatic ectopic ureteroceles, who were treated with prophylactic antimicrobials. They reported that patients without superior urinary tract obstruction, bladder neck obstruction, or severe complicating VUR were treated with prophylactic antimicrobial therapy and follow‐up observation. None of their patients transitioned to surgery during an average 5‐year follow‐up period; five showed HDN improvements.^7^

Our results suggest that the Churchill classification^4^ is useful for selecting cases for conservative treatment. In the Churchill classification, Grade I is defined as those cases where the “[s]uperior kidney of the ureterocele‐affected kidney exhibits mild HDN or mild (Grade I or II) VUR, but the ipsilateral inferior kidney and contralateral kidney are healthy.” Grade II is where the “superior and inferior kidney affected by ureterocele has severe HDN or advanced VUR (Grades III, IV, or V), but the contralateral kidney is healthy.” Grade III is where “both the superior and inferior kidney affected by ureterocele and the contralateral kidney both have advanced HDN and advanced VUR.” According to this classification, our patient 1 was Grade II and patient 2 was Grade I. William *et al*. also used the Churchill classification to calculate asymptomatic ectopic ureterocele outcomes.^8^ As shown in the table, TUI has a success rate of 56% for Grade I and 20% for Grade II patients, and the success rate of the first surgery is 73% for Grade I and 48% for Grade II. Therefore, use of an invasive treatment as the first choice for asymptomatic ectopic ureteroceles in neonates should be considered with caution.

We also investigated eight patients, including past reports and our own experiences, where conservative treatment was successful for asymptomatic ectopic ureteroceles (Table 1). Our investigation suggested that these eight patients were all Churchill classification ≤Grade II, implying that conservative treatment is highly likely to be successful in patients with Churchill classifications of ≤Grade II and who have (i) a nonfunctional superior kidney (superior renal function <10%), (ii) mild HDN of the inferior kidney, or moderate or less VUR, and (iii) no bladder neck obstruction during voiding.

**Table 1:** Conservative ureterocele management and outcomes

Study	Follow‐up	HDN grade at the first visit	HDN grade at the last visit	VUR grade at the first visit	VUR grade at the last visit	Churchill classification
Dirrenna *et al*.^7^	5 years	2	0	III	None	II
Dirrenna *et al*.^7^	5 years	2	0	III	III	II
Dirrenna *et al*.^7^	5 years	2	1	None	None	I
Dirrenna *et al*.^7^	5 years	2	0	III	None	II
Dirrenna *et al*.^7^	5 years	2	2	III	I	II
Dirrenna *et al*.^7^	5 years	1	0	None	None	I
My case 1	3 years 8 months	2	0	IV	III	II
My case 2	4 years 1 month	2	1	II	I	II

HDN grade is as per the Society for Fetal Urology criteria. VUR grade is as per the International Classification of Vesicoureteral Reflux criteria.

Despite the fact that conservative treatment succeeded in the present cases, the natural history of ectopic ureteroceles is unknown, and there are reports of transitions to surgical treatment occurring despite the absence of problems for a certain length of time during conservative follow‐up. Therefore, the exact criteria for selecting patients for conservative treatment are currently unknown.

In conclusion, we reported our experience with the selection of two patients with asymptomatic ectopic ureterocele where conservative treatment was successful, resulting in clear ureterocele reductions. Furthermore, we believe the Churchill classification is useful for selecting patients for conservative treatment. In future instances of asymptomatic ectopic ureterocele, the possibility of implementing conservative treatment should be considered. This requires confirming that the ureterocele does not obstruct the bladder neck and that VUR is mild. If cases are selected appropriately, asymptomatic ectopic ureterocele cases can be treated without invasive treatment.

## Conflict of interest

The authors declare no conflict of interest.
